# Associations between obesity, a composite risk score for probable long COVID, and sleep problems in SARS-CoV-2 vaccinated individuals

**DOI:** 10.1038/s41366-024-01556-w

**Published:** 2024-06-07

**Authors:** Pei Xue, Ilona Merikanto, Eva A. Delale, Adrijana Bjelajac, Juliana Yordanova, Rachel N. Y. Chan, Maria Korman, Sérgio A. Mota-Rolim, Anne-Marie Landtblom, Kentaro Matsui, Catia Reis, Thomas Penzel, Yuichi Inoue, Michael R. Nadorff, Brigitte Holzinger, Charles M. Morin, Colin A. Espie, Giuseppe Plazzi, Luigi De Gennaro, Frances Chung, Bjørn Bjorvatn, Yun Kwok Wing, Yves Dauvilliers, Markku Partinen, Christian Benedict

**Affiliations:** 1 Department of Pharmaceutical Biosciences, Uppsala University, Uppsala, Sweden; 2 Research Programs Unit, Faculty of Medicine, University of Helsinki, Helsinki, Finland; 3 Institute for Anthropological Research, Zagreb, Croatia; 4 Institute for Medical Research and Occupational Health, Zagreb, Croatia; 5 Institute of Neurobiology, Bulgarian Academy of Sciences, Sofia, Bulgaria; 6 Li Chiu Kong Family Sleep Assessment Unit, Department of Psychiatry, Faculty of Medicine, The Chinese University of Hong Kong, Hong Kong SAR, China; 7 Department of Occupational Therapy, Faculty of Health Sciences, Ariel University, Ariel, Israel; 8 Brain Institute, Federal University of Rio Grande do Norte, Federal, Brazil; 9 Department of Medical Sciences, Uppsala University, Uppsala, Sweden; 10 Department of Biomedical and Clinical Sciences, Faculty of Medicine and Health Sciences, Linköping University, Linköping, Sweden; 11 Department of Clinical Laboratory, National Center Hospital, National Center of Neurology and Psychiatry, Tokyo, Japan; 12 Instituto de Medicina Molecular João Lobo Antunes, Faculdade de Medicina, Universidade de Lisboa, Lisbon, Portugal; 13 Faculdade de Ciências Humanas, Universidade Católica Portuguesa, Lisbon, Portugal; 14 Sleep Medicine Center, Charite University Hospital Berlin, Berlin, Germany; 15 Department of Somnology, Tokyo Medical University, Tokyo, Japan; 16 Japan Somnology Center, Institute of Neuropsychiatry, Tokyo, Japan; 17 Department of Psychology, Mississippi State University, Mississippi, MI USA; 18 Department of Psychiatry and Behavioral Sciences, Baylor College of Medicine, Baylor, TX USA; 19 Medical University of Vienna, Postgraduate, Schlafcoaching, Vienna, Austria; 20 Centre de recherche CERVO/Brain Research Center, École de psychologie, Université Laval, Quebec City, Quebec, Canada; 21 Sleep and Circadian Neuroscience Institute, Nuffield Department of Clinical Neurosciences, University of Oxford, Oxford, QC UK; 22 IRCCS Istituto Delle Scienze Neurologiche di Bologna, Bologna, Italy; 23 Department of Biomedical, Metabolic and Neural Sciences, University of Modena and Reggio Emilia, Modena, Italy; 24 Department of Psychology, Sapienza University of Rome, Roma, Lazio Italy; 25 IRCCS Fondazione Santa Lucia, Roma, Italy; 26 Department of Anesthesiology and Pain Medicine, University Health Network, University of Toronto, Toronto, ON Canada; 27 Department of Global Public Health and Primary Care, University of Bergen, Bergen, Norway; 28 Norwegian Competence Center for Sleep Disorders, Haukeland University Hospital, Bergen, Norway; 29 Sleep-Wake Disorders Unit, Department of Neurology, Gui-de-Chauliac Hospital, CHU Montpellier, France; 30 INM, University Montpellier, INSERM, Montpellier, France; 31 Department of Clinical Neurosciences, University of Helsinki Clinicum Unit, Helsinki, Finland; 32 Helsinki Sleep Clinic, Terveystalo Healthcare Services, Helsinki, Finland

**Keywords:** Epidemiology, Immunological disorders

## Abstract

**Background:**

Preliminary data suggests that obesity might hasten the decline in mRNA vaccine-induced immunity against SARS-CoV-2. However, whether this renders individuals with obesity more susceptible to long COVID symptoms post-vaccination remains uncertain. Given sleep’s critical role in immunity, exploring the associations between obesity, probable long COVID symptoms, and sleep disturbances is essential.

**Methods:**

We analyzed data from a survey of 5919 adults aged 18 to 89, all of whom received two SARS-CoV-2 mRNA vaccinations. Participants were categorized into normal weight, overweight, and obesity groups based on ethnicity-specific BMI cutoffs. The probability of long COVID was evaluated using the Post-Acute Sequelae of SARS-CoV-2 (PASC) score, as our survey did not permit confirmation of acute SARS-CoV-2 infection through methods such as antibody testing. Additionally, sleep patterns were assessed through questionnaires.

**Results:**

Participants with obesity exhibited a significantly higher adjusted odds ratio (OR) of having a PASC score of 12 or higher, indicative of probable long COVID in our study, compared to those with normal weight (OR: 1.55, 95% CI: 1.05, 2.28). No significant difference was observed for overweight individuals (OR: 0.92 [95% CI: 0.63, 1.33]). Both obesity and probable long COVID were associated with increased odds of experiencing a heightened sleep burden, such as the presence of obstructive sleep apnea or insomnia (*P* < 0.001). However, no significant interaction between BMI and probable long COVID status was found.

**Conclusions:**

Even post-vaccination, individuals with obesity may encounter a heightened risk of experiencing prolonged COVID-19 symptoms. However, confirming our observations necessitates comprehensive studies incorporating rigorous COVID infection testing, such as antibody assays - unavailable in our anonymous survey. Additionally, it is noteworthy that the correlation between probable long COVID and sleep disturbances appears to be independent of BMI.

## Introduction

According to the World Health Organization, since December 2019, over 760 million humans have contracted the severe acute respiratory syndrome coronavirus 2 (SARS-CoV-2), resulting in Coronavirus Disease 2019 (COVID-19), with a recorded global death toll of 6.9 million attributed to the infection [1]. A meta-analysis indicates that approximately 43% of individuals diagnosed with COVID-19 have reported experiencing lingering symptoms or health issues following the acute phase of infection, also named long COVID [2]. Numerous medications have been introduced to mitigate the severe and long-lasting health effects associated with COVID-19, including antiviral agents such as remdesivir [3]. However, vaccinations against SARS-CoV-2, particularly utilizing the recently developed mRNA vaccines, have emerged as the most potent measure in combating the virus [4]. For instance, mRNA vaccines have been associated with a reduced likelihood of contracting SARS-CoV-2, being hospitalized, or dying from COVID-19 [5]. Furthermore, they may lower the risk of experiencing long COVID symptoms, such as chest pain, breathing difficulties, ageusia, or anosmia [6].

People with obesity face an increased risk of developing a more severe course of COVID-19. For instance, in a study of COVID-19 cases among patients aged 18 years and younger, individuals with obesity had a 3.1 times higher risk of hospitalization and a 1.4 times higher risk of severe illness when hospitalized, which includes admission to the intensive care unit, need for invasive mechanical ventilation, or death [7]. Moreover, obesity has been identified as a risk factor for persistent symptoms following SARS-CoV-2 infection [8–11]. Given that mRNA vaccination against SARS-CoV-2 reduces both mortality and morbidity of COVID-19 [4, 5], including long COVID [6], it may be particularly relevant for people with obesity. However, a recent study suggests that two doses of SARS-CoV-2 mRNA vaccine may provide less protection in people with obesity. In this study, it was observed that breakthrough infections occurred more frequently and developed more rapidly in individuals with obesity who had received two doses of mRNA vaccine, compared to those with normal weight [12]. However, it remains unclear whether this weight-related effect also influences the risk of experiencing long COVID symptoms in individuals who have received two doses of the SARS-CoV-2 mRNA vaccine.

In our present multinational anonymous survey study, we included 5919 adults who had completed a two-dose regimen of SARS-CoV-2 mRNA vaccination. Our primary objective was to investigate whether individuals with obesity have a higher odds ratio (OR) of experiencing symptoms resembling long COVID, thus rejecting the null hypothesis that BMI status does not affect the likelihood of experiencing such symptoms. Given that only a small proportion of respondents reported a positive COVID test result, the assessment of long COVID likelihood relied on the Post-Acute Sequelae of SARS-CoV-2 (PASC) score [13]. This scoring system incorporates various typical symptoms associated with long COVID to ascertain its presence. Additionally, we explored the potential impact of gender on the relationship between BMI status and long COVID-like symptoms, considering gender-based differences in long COVID risk [14].

Notably, research indicates a link between obesity and sleep, encompassing issues such as insomnia [15], obstructive sleep apnea (OSA; [16]), and presence of short sleep duration [17]. Given sleep’s pivotal role in managing viral infections [18, 19], including SARS-CoV-2 [20–23], it is reasonable to hypothesize that individuals with obesity may be more prone to experiencing symptoms akin to long COVID compared to those with normal weight, owing to heightened sleep burden. Consequently, we investigated whether the occurrence of insomnia, OSA, and both short and long sleep durations is more prevalent among participants with probable long COVID and obesity.

## Study design and statistical methods

### Study population

In this study, self-reported data were collected from 5919 participants aged 18 to 89, with 63% of participants being women. The data were obtained through the second wave of the **I**nternational **CO**VID **S**leep **S**tudy survey (ICOSS-2), as described in detail in reference [24]. The survey was conducted anonymously between May and December 2021 and was accessible through various online platforms, including RedCap and Qualtrics. To reach a diverse audience, the survey was advertised on university web pages, newspapers, television, Facebook, and Twitter (since 2023 named X). Additionally, it was made available in multiple languages, including German, Portuguese, Brazilian Portuguese, English, French, Bulgarian, Croatian, Chinese, Finnish, Hebrew, Italian, Japanese, Norwegian, and Swedish. After applying the exclusion criteria outlined in Supplemental Table S1, 5919 participants who reported receiving two doses of mRNA SARS-CoV-2 vaccine (Moderna and BioNTech/Pfizer) had complete data for analysis. During the survey period in 2021, a third dose of SARS-CoV-2 mRNA vaccine was not yet standard. Therefore, we only inquired about participants receiving up to two doses of SARS-CoV-2 mRNA vaccine.

This study adhered to the principles outlined in the Helsinki Declaration and obtained either ethical approval or waivers in all participating countries, in accordance with their respective national research governance and regulations. Notably, ethical approval was not mandated by national law in Austria, Brazil, Finland, France, Norway, and Sweden due to the anonymous nature of the survey collection. The anonymity of the data was preserved throughout the study. Prior to accessing the questionnaire, participants were required to provide their consent to participate, with a minimum age requirement of 18 years. No monetary compensation was provided to participants. Further details regarding ethical approval in each country can be found in Supplemental Table S2.

### Definition of BMI cut-off points

The BMI ranges used to define normal weight, overweight, and obesity vary for Asians compared to other ethnicities [25]. Hence, we employed the following BMI thresholds for non-Asians: normal weight (reference group) <25 kg/m^2^, overweight 25-29.9 kg/m^2^, and obesity ≥30 kg/m^2^. Meanwhile, for participants of Asian ethnicity, their BMI status was determined as follows: normal weight <23 kg/m^2^, overweight ≥23 to <27.5 kg/m^2^, and obesity ≥27.5 kg/m^2^.

### Definition of composite risk score for long COVID

A recent study [13] identified 12 symptoms that persist for at least six months post-infection with SARS-CoV-2 that can be used to assess the likelihood of experiencing long COVID, using a PASC score. Specifically, each symptom was assigned a score based on its predictive ability for long COVID: loss of or change in smell or taste (8 points), post-exertional malaise (7 points), chronic cough (4 points), brain fog (3 points), thirst (3 points), palpitations (2 points), chest pain (2 points), fatigue (1 point), dizziness (1 point), gastrointestinal tract symptoms (1 point), changes in sexual desire or capacity (1 point), and abnormal movements (1 point).

We included eight symptoms that lasted for at least six months at the time of the survey to calculate the PASC score for each participant. These symptoms were: loss of or change in smell or taste, post-exertional malaise, brain fog, palpitations, chest pain, fatigue, dizziness, and gastrointestinal tract symptoms. Based on a previous study’s proposal [13], participants with a PASC score of 12 or higher were classified as having a high likelihood of experiencing long COVID, regardless of whether they reported a previous SARS-CoV-2 infection.

To address the potential bias of misclassifying participants as unlikely to suffer from probable long COVID due to the absence of survey questions about chronic cough, thirst, changes in sexual desire or capacity, and abnormal movements, a sensitivity analysis was performed (see statistical section for more details).

### Assessment of sleep

Based on participants’ sleep duration reports, we evaluated if participants usually slept less than six or more than nine hours per night (in the following, referred to as short or long sleep duration, respectively). The literature often uses these thresholds to discriminate short and long sleep duration from normal sleep duration [26, 27]. Participants’ insomnia risk was determined through the Insomnia Severity Index (ISI) [28], a validated questionnaire of seven items assessing the severity of insomnia symptoms and their impact on daily functioning. A score greater than 14 is indicative of moderate-to-severe insomnia. To assess the presence of OSA, we used the STOP scale [29]. Specifcally, participants were asked to respond on a 5-point Likert scale to the following four questions: (a) Do you snore loudly, surpassing the volume of talking or being audible through closed doors? Response options ranged from “Not at all” to “Every night/almost every night.” (b) Do you frequently experience daytime tiredness, fatigue, or excessive sleepiness? Response options spanned from “Not at all” to “Every day/almost every day.” (c) Has anyone ever witnessed you ceasing to breathe or choking during your sleep? Response options varied from “No, never” to “Every day/almost every day.” Participants’ answers were dichotomized into two categories for the first three questions: 0 = Less than three nights per week and 1 = Three nights per week or more. (d) We also surveyed whether participants currently had or received treatment for high blood pressure. A “Yes”-response was counted as one score. The risk of OSA was considered high if participants scored two or greater on the STOP scale.

### Statistical analysis

Data are presented as mean ± SD unless otherwise specified. Group characteristics were compared using the Chi-Square test for categorical variables and generalized linear models for continuous variables. Logistic regression analyses were conducted to examine the associations between BMI group as a predictor and probable long COVID status as a dependent variable using SPSS 28.0 (IBM Corp., Armonk, NY, USA).

In addition to conducting an unadjusted logistic regression analysis, we employed one additional regression model to examine the robustness of the association between BMI status and probable long COVID. The adjusted analysis incorporated self-reported positive SARS-CoV-2 test, age, sex, race/ethnicity, smoking status, the time elapsed since the first vaccination ( ≤ six vs. > six months), urbanicity, and weekly physical activity level score (ranging from 0 to 7; higher score indicating higher physical activity; for more details, see [22]). We additionally considered a medical history encompassing hypertension, type 2 diabetes, depression, and attention deficit hyperactivity disorder. These were defined as instances where individuals had been diagnosed with or received treatment for these conditions either prior to or at the time of the survey. This inclusion was motivated by the recognition that each of these conditions commonly co-occurs with obesity [30–33].

To ensure the robustness of the hypothesized association between BMI status and probable long COVID, we conducted several sensitivity analyses:

1. We examined individuals who reported testing positive for SARS-CoV-2 before the survey (n = 515).

2. We separately analyzed data for men and women because previous findings suggest that the risk of long COVID is higher among women than men [14]. In this context, we assessed multiplicative interactions between BMI status and sex.

3. Participants from the USA were excluded from the analysis as they were significantly younger than participants from other countries (Supplemental Table S3).

4. We excluded individuals whose PASC score fell between 3 and 11, as our study did not survey four of the twelve symptoms used to calculate the PASC score in a previous study [13]. As mentioned earlier, if experienced for at least six months, the total sum of these symptoms corresponds to 9 points.

5. We excluded participants (n = 412) who were underweight from the normal weight reference group. Underweight was defined as having a BMI < 18.5 kg/m^2^ across all ethnicities.

To assess the potential variability in the risk of inadequate sleep associated with BMI and probable long COVID status, we conducted logistic regression analyses, both unadjusted and adjusted. Our binary outcome variables included moderate-to-severe insomnia, a high risk of OSA, short nighttime sleep duration (less than 6 hours), and long nighttime sleep duration (more than 9 hours). In the adjusted regression model, both BMI group and probable long COVID status were entered together to account for mutual adjustment. Additionally, for the sleep outcomes, we assessed multiplicative interactions between BMI group and probable long COVID status. Overall, a *P* value less than 0.05 was considered significant.

## Results

### Cohort characteristics

A comprehensive overview of characteristics, categorized by BMI status, is provided in Table 1. Compared to participants with normal weight, those with obesity exhibited several distinctive features. Specifically, they were more likely to be White/Caucasian and reside in urban areas. Additionally, they were more likely to report a history of type 2 diabetes, hypertension, depression, and attention deficit hyperactivity disorder. Additionally, they more frequently met the criteria for OSA and moderate-to-severe insomnia and reported shorter sleep durations. Finally, participants in the obesity group had significantly higher PASC scores, indicating a greater likelihood of experiencing persistent symptoms commonly associated with a prior COVID-19 infection. The participant’s country of origin is specified in Supplemental Table S3.

**Table 1:** Characteristics of ICOSS-2 participants categorized by body mass index status (*N* = 5919).

Variable	Normal weight	Overweight	Obesity	*P* value
Participants, total number	3406	1710	803	**--**
PASC score, mean (SD)	1.0 (3.0)	1.2 (3.6)	2.9 (5.2)	<0.001
PASC score ≥12	93 (2.7)	58 (3.4)	74 (9.2)	<0.001
Age (years), mean (SD)	43.2 (17.7)	48.4 (16.4)	47.5 (15.8)	<0.001
Sex				<0.001
Male	1097 (32.2)	828 (48.4)	294 (36.6)	
Female	2309 (67.8)	882 (51.6)	509 (63.4)	
Race/ethnicity				<0.001
White/Caucasian	1580 (46.4)	837 (48.9)	535 (66.6)	
Asian	1557 (45.7)	757 (44.3)	201 (25.0)	
Other	269 (7.9)	116 (6.8)	67 (8.3)	
Smoking status				0.19
Never or less than once per month	2736 (80.3)	1351 (79.0)	633 (78.8)	
Less than once per week	88 (2.6)	52 (3.0)	15 (1.9)	
1–5 days per week	96 (2.8)	38 (2.2)	19 (2.4)	
Every day or almost daily	486 (14.3)	269 (15.7)	136 (16.9)	
Weekly physical activity score, mean (SD)	1.9 (2.4)	1.9 (2.3)	1.5 (2.1)	<0.001
Urbanicity	1871 (54.9)	976 (57.1)	525 (65.4)	<0.001
≤6 months have elapsed since the first mRNA vaccination	2818 (82.7)	1336 (78.1)	648 (80.7)	<0.001
Body mass index (kg/m^2^), mean (SD)	21.0 (2.1)	26.0 (1.7)	33.4 (3.7)	<0.001
Reported a positive SARS-CoV-2 test result	270 (7.9)	145 (8.5)	100 (12.5)	<0.001
Regularly sleeping less than 6 hours per night	474 (13.9)	314 (18.4)	180 (22.4)	<0.001
Regularly sleeping more than 9 hours per night	97 (2.8)	69 (4.0)	25 (3.1)	0.08
Insomnia severity index score, mean (SD)	7.5 (5.6)	8.3 (5.9)	10.1 (6.8)	<0.001
High risk for moderate-to-severe insomnia	420 (12.3)	279 (16.3)	230 (28.6)	<0.001
High risk for obstructive sleep apnea	119 (3.5)	159 (9.3)	166 (20.7)	<0.001
History of type 2 diabetes	75 (2.2)	72 (4.2)	64 (8.0)	<0.001
History of hypertension	299 (8.8)	357 (20.9)	272 (33.9)	<0.001
History of attention deficit hyperactivity disorder	271 (8.0)	152 (8.9)	118 (14.7)	<0.001
History of depression	341 (10.0)	235 (13.7)	217 (27.0)	<0.001

Data are presented as numbers (percentages) unless otherwise stated. Group comparisons were conducted using Chi-Square tests for categorical variables and generalized linear models for continuous variables (where *P* values refer to the main effect of BMI status). A significance level of *P* < .05 was used to determine statistical significance. *PASC* post-acute sequelae of SARS-CoV-2.

### Association between obesity and long COVID status

**Fig. 1:**
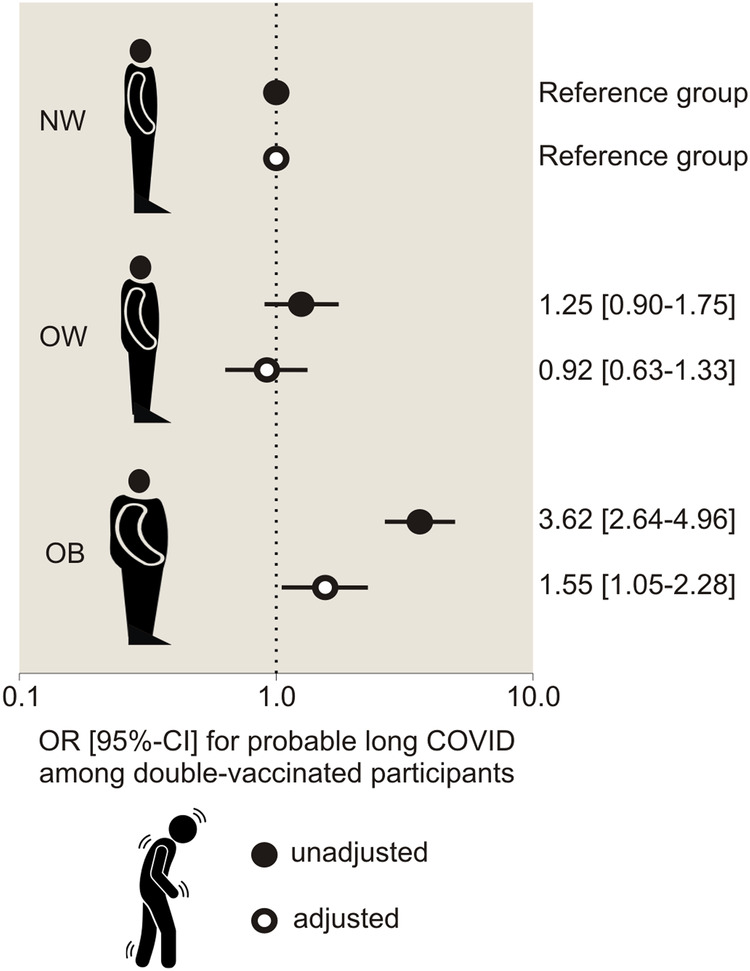
Association of body mass index with probable long COVID status. OR [95% CI] indicates odds ratio [95% Confidence Interval]. NW participants with normal weight, OW participants with overweight; and OB, participants with obesity.

The frequency of PASC symptoms among double-vaccinated ICOSS-2 participants, categorized by BMI group alone, as well as by combined BMI group and probable long COVID status, is presented in Supplemental Table S4 and Supplemental Table S5, respectively. As depicted in Fig. 1, participants with obesity had 1.55 times higher OR of having a PASC score ≥12 [95% CI: 1.05, 2.28], compared to those with normal weight (adjusted *P* = 0.028). However, there were no significant differences in OR of having a PASC score ≥12 between participants with overweight and those with normal weight, both in the unadjusted (*P* = 0.188) and the adjusted analyses (*P* = 0.650; Fig. 1).

In the subgroup analysis of ICOSS-2 participants who indicated that they had tested positive for SARS-CoV-2 before the survey, the unadjusted OR of having a PASC score ≥12 was 2.61 times higher in participants with obesity (n = 100) compared to the normal weight reference group (n = 270; [95% CI: 1.49, 4.58], *P* < 0.001). However, the unadjusted OR of having a PASC score ≥12 between participants with overweight (n = 145) and those with normal weight did not significantly differ (OR: 1.01 [95% CI: 0.56, 1.84], *P* = 0.968). After adjusting for potential confounders, the OR of having a PASC score ≥12 did not significantly differ among the BMI groups (*P* = 0.808 for obesity vs. normal weight and *P* = 0.381 for overweight vs. normal weight, respectively).

As summarized in Supplemental Table S6, the association between obesity and a having a PASC score ≥12 was present in both men and women in the unadjusted analysis (*P* < 0.001). Following adjustment, OR of having a PASC score ≥12 were only significantly higher among women (adjusted *P* = 0.032). However, no significant multiplicative interaction between participants’ sex and BMI status was found (adjusted *P* = 0.518).

Ultimately, the correlation between obesity and an increased OR of having a PASC score ≥12 remained significant even after excluding participants from the USA (Supplemental Table S7), those from the group without probable long COVID whose PASC score ranged from 3 to 11 (Supplemental Table S8), and individuals who were underweight (Supplemental Table S9).

### Association between obesity, probable long COVID, and inadequate sleep

As presented in Table 2, a significant correlation between overweight and obesity and elevated adjusted ORs ranging from 1.33 to 5.12 [95% CI range: 1.11, 6.70] for experiencing moderate-to-severe insomnia, OSA, and short sleep duration was found (adjusted *P* ≤ 0.002). Furthermore, participants with obesity had lower OR of sleeping more than 9 h (OR: 0.60 [95% CI: 0.37, 0.98], *P* = 0.042). Additionally, irrespective of their BMI status, participants with probable long COVID exhibited a heightened likelihood of experiencing moderate-to-severe insomnia, OSA, and prolonged sleep duration (adjusted ORs ranging from 1.98 to 2.88 [95% CI range: 1.32, 4.78]; adjusted *P* ≤ 0.008).

**Table 2:** Association between probable long COVID, body mass index, and sleep in ICOSS-2.

Group status	OR [95% CI] for being at high risk for moderate-to-severe insomnia		OR [95% CI] for having a high risk for obstructive sleep apnea		OR [95% CI] for sleeping regularly less than 6 hours per night		OR [95% CI] for sleeping regularly more than 9 hours per night	
	unadjusted	adjusted^a^	unadjusted	adjusted^a,b^	unadjusted	adjusted^a^	unadjusted	adjusted^a^
Normal weight (N = 3406)	1	1	1	1	1	1	1	1
Overweight (N = 1710)	1.39 [1.18–1.63]	1.33 [1.11–1.59]	2.83 [2.22–3.62]	2.26 [1.75–2.91]	1.39 [1.19–1.63]	1.35 [1.15–1.59]	1.43 [1.05–1.96]	1.33 [0.96–1.86]
Obesity (N = 803)	2.85 [2.37–3.43]	1.83 [1.48–2.26]	7.20 [5.61–9.25]	5.12 [3.91–6.70]	1.79 [1.48–2.17]	1.63 [1.32–2.00]	1.10 [0.70–1.71]	0.60 [0.37–0.98]
PASC score <12 (N = 5964)	1	1	1	1	1	1	1	1
PASC score ≥12 (N = 225)	6.15 [4.69–8.07]	2.26 [1.64–3.10]	4.18 [3.02–5.78]	1.98 [1.32–2.96]	1.65 [1.21–2.26]	1.06 [0.75–1.51]	4.82 [3.15–7.38]	2.88 [1.73–4.78]

*PASC* post-acute sequelae SARS-CoV-2.

^a^Independent variables included in the adjusted logistic regression model: body mass index status, probable long COVID status, age, sex, ethnicity, SARS-CoV-2 test positivity report, the time elapsed since first vaccination, smoking status, living area, weekly physical activity level status score, presence of hypertension, presence of type 2 diabetes, presence of attention deficit hyperactivity disorder, and presence of depression.

^b^The adjusted logistic regression model did not contain hypertension as an independent variable, as it is a component of the STOP score used to assess the risk for obstructive sleep apnea [29].

A separate logistic regression analysis, incorporating the multiplicative interaction term between BMI and probable long COVID status, indicated that the association between participants’ probable long COVID status and sleep outcomes remained consistent across different BMI statuses (adjusted *P* ≥ 0.141 for the multiplicative interaction between BMI and probable long COVID status).

## Discussion

In our international survey study, encompassing 5919 participants aged 18 to 89 years who had received two doses of SARS-CoV-2 mRNA vaccine, we found that participants with obesity exhibited a higher likelihood of experiencing multiple probable long COVID symptoms, as determined by a composite risk score for long COVID, compared to those with normal weight. This finding challenges the null hypothesis, indicating that BMI status indeed influences the probability of experiencing long COVID symptoms.

While our survey study did not directly investigate the mechanisms underlying the association between obesity and experiencing probable long COVID, despite being double-vaccinated with SARS-CoV-2 mRNA vaccine, several potential explanations exist. For instance, a previous study [12] revealed that vaccinated participants with severe obesity had a 76% higher likelihood of experiencing hospitalization or death from COVID-19. This finding may be attributed to the fact that 55% of participants with severe obesity had undetectable levels of neutralizing antibodies against SARS-CoV-2, compared to only 12% of those with normal BMI six months after their second vaccine dose. Additionally, the neutralizing capacity of SARS-CoV-2-specific antibodies was lower in participants with obesity compared to those with a BMI in the normal weight range.

In our study, participants with probable long COVID exhibited elevated odds of experiencing sleep disorders, including moderate-to-severe insomnia and a high risk for OSA, as well as prolonged sleep durations, compared to participants with a low likelihood of long COVID. It is noteworthy that participants with obesity presented with a heightened sleep burden, manifesting as a higher likelihood of suffering from insomnia, OSA, and short sleep duration. However, associations between sleep outcomes and probable long COVID did not vary by BMI status. This suggests that although improving sleep in patients with persistent COVID symptoms may hold promise for reducing the frequency, duration, and severity of such symptoms - given that sleep enhances innate and adaptive immunity [18, 19, 34] - the heightened sleep burden due to probable long COVID may occur regardless of a participant’s BMI status.

### Limitations

When interpreting our results, it is important to consider several factors. Firstly, we relied on self-reported data in this study, which could have introduced recall bias. Secondly, to mitigate the risk of misclassifying participants experiencing potential long COVID symptoms, our survey study utilized the PASC score, incorporating a cluster of post-COVID infection symptoms documented in the literature [13]. However, concerns remain regarding participants with a PASC score of 12 or higher, especially if previous COVID infection has not been confirmed, as this could stem from conditions beyond those considered in our analysis. Thus, comprehensive studies, which include rigorous testing for COVID infections such as antibody assays and polymerase chain reaction testing, are necessary to confirm our findings. Another limitation is that various factors such as limited access to SARS-CoV-2 tests, timing issues related to test sensitivity, and reluctance among respondents to undergo testing may contribute to the fact that only 9% of participants reported a positive SARS-CoV-2 result. Additionally, previous research has highlighted that a significant number of SARS-CoV-2 cases go undetected [35], with some individuals potentially unaware of their infection. It is also worth mentioning that our analysis focused exclusively on SARS-CoV-2 mRNA vaccine, as the majority of respondents received this type of vaccine. Finally, future studies could enhance their comprehensiveness by incorporating additional long COVID symptoms, as demonstrated in previous research [36]. This approach would contribute to a deeper understanding of how BMI status may influence both the risk and severity of long COVID symptoms despite vaccination against SARS-CoV-2.

## Conclusions

Participants with obesity may face an increased likelihood of experiencing multiple symptoms attributed to long COVID, even after receiving two doses of the SARS-CoV-2 mRNA vaccine, compared to individuals with normal weight. This finding from our study aligns well with previous results suggesting that participants with obesity exhibit a faster decline in immunity against SARS-CoV-2 following vaccination compared to those with normal weight [12]. The association between probable long COVID and disrupted sleep, irrespective of BMI, may be significant from a therapeutic standpoint, as sleep can enhance immunity [18, 19, 34, 37] and thus aid in the recovery from long COVID. However, given the limitations of our study, such as reliance on self-reported data and potential confounding factors, our findings should be viewed as hypothesis-generating rather than definitive conclusions.

### Supplementary information

Supplementary Information: Associations Between Obesity, a Composite Risk Score for Probable Long COVID, and Sleep Problems in SARS-CoV-2 Vaccinated Individuals

## Data Availability

Raw data from the ICOSS surveys are accessible to researchers affiliated with universities upon approval from the ICOSS core group. For inquiries, please contact Bjørn Bjorvatn at Bjorn.Bjorvatn@uib.no.

## References

[1] Coronavirus disease (COVID-19). https://www.who.int/news-room/fact-sheets/detail/coronavirus-disease-(covid-19); date of retrieval: 24/05/24

[2] Chen C, Haupert SR, Zimmermann L, Shi X, Fritsche LG, Mukherjee B. Global prevalence of post-Coronavirus Disease 2019 (COVID-19) condition or long COVID: a meta-analysis and systematic review. J Infect Dis. 2022;226:1593–607. 35429399 10.1093/infdis/jiac136 PMC9047189

[3] Gottlieb RL, Vaca CE, Paredes R, Mera J, Webb BJ, Perez G, et al. Early remdesivir to prevent progression to severe Covid-19 in outpatients. N Engl J Med. 2022;386:305–15. 34937145 10.1056/NEJMoa2116846 PMC8757570

[4] Doria-Rose N, Suthar MS, Makowski M, O’Connell S, McDermott AB, Flach B, et al. Antibody persistence through 6 months after the second dose of mRNA-1273 vaccine for Covid-19. N Engl J Med. 2021;384:2259–61. 33822494 10.1056/NEJMc2103916 PMC8524784

[5] Mohammed I, Nauman A, Paul P, Ganesan S, Chen KH, Jalil SMS, et al. The efficacy and effectiveness of the COVID-19 vaccines in reducing infection, severity, hospitalization, and mortality: a systematic review. Hum Vaccin Immunother. 2022;18:2027160. 35113777 10.1080/21645515.2022.2027160 PMC8862168

[6] Ayoubkhani D, Bermingham C, Pouwels KB, Glickman M, Nafilyan V, Zaccardi F, et al. Trajectory of long covid symptoms after covid-19 vaccination: community based cohort study. BMJ. 2022;377:e069676. 35584816 10.1136/bmj-2021-069676 PMC9115603

[7] Kompaniyets L, Agathis NT, Nelson JM, Preston LE, Ko JY, Belay B, et al. Underlying Medical Conditions Associated With Severe COVID-19 Illness Among Children. JAMA Netw Open. 2021;4:e2111182. 34097050 10.1001/jamanetworkopen.2021.11182 PMC8185607

[8] Luo J, Zhang J, Tang HT, Wong HK, Lyu A, Cheung CH, et al. Prevalence and risk factors of long COVID 6-12 months after infection with the Omicron variant among nonhospitalized patients in Hong Kong. J Med Virol. 2023;95:e28862. 37334978 10.1002/jmv.28862

[9] Loosen SH, Jensen BO, Tanislav C, Luedde T, Roderburg C, Kostev K. Obesity and lipid metabolism disorders determine the risk for development of long COVID syndrome: a cross-sectional study from 50,402 COVID-19 patients. Infection. 2022;50:1165–70. 35355237 10.1007/s15010-022-01784-0 PMC8966865

[10] Heubner L, Petrick PL, Güldner A, Bartels L, Ragaller M, Mirus M, et al. Extreme obesity is a strong predictor for in-hospital mortality and the prevalence of long-COVID in severe COVID-19 patients with acute respiratory distress syndrome. Sci Rep. 2022;12:18418. 36319681 10.1038/s41598-022-22107-1 PMC9626466

[11] Vimercati L, De Maria L, Quarato M, Caputi A, Gesualdo L, Migliore G, et al. Association between long COVID and overweight/obesity. J Clin Med. 2021;10:4143. 34575251 10.3390/jcm10184143 PMC8469321

[12] van der Klaauw AA, Horner EC, Pereyra-Gerber P, Agrawal U, Foster WS, Spencer S, et al. Accelerated waning of the humoral response to COVID-19 vaccines in obesity. Nat Med. 2023;29:1146–54. 37169862 10.1038/s41591-023-02343-2 PMC10202802

[13] Thaweethai T, Jolley SE, Karlson EW, Levitan EB, Levy B, McComsey GA, et al. Development of a definition of postacute sequelae of SARS-CoV-2 Infectio. JAMA. 2023;329:1934–1946. 37278994 10.1001/jama.2023.8823 PMC10214179

[14] Davis HE, McCorkell L, Vogel JM, Topol EJ. Long COVID: major findings, mechanisms and recommendations. Nat Rev Microbiol. 2023;21:133–46. 36639608 10.1038/s41579-022-00846-2 PMC9839201

[15] Pearson NJ, Johnson LL, Nahin RL. Insomnia, trouble sleeping, and complementary and alternative medicine: Analysis of the 2002 national health interview survey data. Arch Intern Med. 2006;166:1775–82. 16983058 10.1001/archinte.166.16.1775

[16] Gami AS, Caples SM, Somers VK. Obesity and obstructive sleep apnea. Endocrinol Metab Clin North Am. 2003;32:869–94. 14711066 10.1016/S0889-8529(03)00069-0

[17] van den Berg JF, Knvistingh Neven A, Tulen JH, Hofman A, Witteman JC, Miedema HM, et al. Actigraphic sleep duration and fragmentation are related to obesity in the elderly: the Rotterdam Study. Int J Obes (Lond). 2008;32:1083–90. 18414418 10.1038/ijo.2008.57

[18] Besedovsky L, Lange T, Born J. Sleep and immune function. Pflugers Arch. 2012;463:121–37. 22071480 10.1007/s00424-011-1044-0 PMC3256323

[19] Spiegel K, Rey AE, Cheylus A, Ayling K, Benedict C, Lange T, et al. A meta-analysis of the associations between insufficient sleep duration and antibody response to vaccination. Curr Biol. 2023;33:998–1005.e2. 36917932 10.1016/j.cub.2023.02.017

[20] Izuhara M, Matsui K, Yoshiike T, Kawamura A, Utsumi T, Nagao K, et al. Association between sleep duration and antibody acquisition after mRNA vaccination against SARS-CoV-2. Front Immunol. 2023;14:1242302. 38149250 10.3389/fimmu.2023.1242302 PMC10750410

[21] Lin YN, Zhou LN, Liu ZR, Wang Y, Li SQ, Lu FY, et al. Short sleep duration is associated with prolonged virus shedding in SARS-CoV-2 omicron-infected patients. Nat Sci Sleep. 2023;15:547–54. 37441268 10.2147/NSS.S411677 PMC10335320

[22] Xue P, Merikanto I, Chung F, Morin CM, Espie C, Bjorvatn B, et al. Persistent short nighttime sleep duration is associated with a greater post-COVID risk in fully mRNA-vaccinated individuals. Transl Psychiatry. 2023;13:32. 36726008 10.1038/s41398-023-02334-4 PMC9890416

[23] Athanasiou N, Baou K, Papandreou E, Varsou G, Amfilochiou A, Kontou E, et al. Association of sleep duration and quality with immunological response after vaccination against severe acute respiratory syndrome coronavirus-2 infection. J Sleep Res. 2023;32:e13656. 35670298 10.1111/jsr.13656 PMC9348328

[24] Merikanto I, Dauvilliers Y, Chung F, Wing YK, De Gennaro L, Holzinger B, et al. Sleep symptoms are essential features of long-COVID - Comparing healthy controls with COVID-19 cases of different severity in the international COVID sleep study (ICOSS-II). J Sleep Res. 2023;32:e13754. 36208038 10.1111/jsr.13754 PMC9874584

[25] Expert Consultation WHO. Appropriate body-mass index for Asian populations and its implications for policy and intervention strategies. Lancet. 2004;363:157–63. 14726171 10.1016/S0140-6736(03)15268-3

[26] Pérez-Carbonell L, Mignot E, Leschziner G, Dauvilliers Y. Understanding and approaching excessive daytime sleepiness. Lancet. 2022;400:1033–46. 36115367 10.1016/S0140-6736(22)01018-2

[27] Ohayon MM, Reynolds CF 3rd, Dauvilliers Y. Excessive sleep duration and quality of life. Ann Neurol. 2013;73:785–94. 23846792 10.1002/ana.23818 PMC4142503

[28] Bastien CH, Vallières A, Morin CM. Validation of the Insomnia Severity Index as an outcome measure for insomnia research. Sleep Med. 2001;2:297–307. 11438246 10.1016/S1389-9457(00)00065-4

[29] Chung F, Yegneswaran B, Liao P, Chung SA, Vairavanathan S, Islam S, et al. STOP questionnaire: a tool to screen patients for obstructive sleep apnea. Anesthesiology. 2008;108:812–21. 18431116 10.1097/ALN.0b013e31816d83e4

[30] El Meouchy P, Wahoud M, Allam S, Chedid R, Karam W, Karam S. Hypertension related to obesity: pathogenesis, characteristics and factors for control. Int J Mol Sci. 2022;23:12305. 36293177 10.3390/ijms232012305 PMC9604511

[31] Kahn SE, Hull RL, Utzschneider KM. Mechanisms linking obesity to insulin resistance and type 2 diabetes. Nature. 2006;444:840–6. 17167471 10.1038/nature05482

[32] Lanoye A, Adams E, Fuemmeler BF. Obesity and attention-deficit hyperactivity disorder. Curr Top Behav Neurosci. 2022;57:221–41. 35505058 10.1007/7854_2022_337

[33] Jokela M, Laakasuo M. Obesity as a causal risk factor for depression: systematic review and meta-analysis of Mendelian Randomization studies and implications for population mental health. J Psychiatr Res. 2023;163:86–92. 37207436 10.1016/j.jpsychires.2023.05.034

[34] Christoffersson G, Vågesjö E, Pettersson US, Massena S, Nilsson EK, Broman JE, et al. Acute sleep deprivation in healthy young men: impact on population diversity and function of circulating neutrophils. Brain Behav Immun. 2014;41:162–72. 24878171 10.1016/j.bbi.2014.05.010

[35] Lau H, Khosrawipour T, Kocbach P, Ichii H, Bania J, Khosrawipour V. Evaluating the massive underreporting and undertesting of COVID-19 cases in multiple global epicenters. Pulmonology. 2021;27:110–5. 32540223 10.1016/j.pulmoe.2020.05.015 PMC7275155

[36] Fischer A, Badier N, Zhang L, Elbéji A, Wilmes P, Oustric P, et al. Long COVID classification: findings from a clustering analysis in the predi-COVID cohort study. Int J Environ Res Public Health. 2022;19:16018. 36498091 10.3390/ijerph192316018 PMC9740149

[37] Besedovsky L, Lange T, Haack M. The sleep-immune crosstalk in health and disease. Physiol Rev. 2019;99:1325–180. 30920354 10.1152/physrev.00010.2018 PMC6689741

