# Supplementary material for: Associations between obesity, a composite risk score for probable long COVID, and sleep problems in SARS-CoV-2 vaccinated individuals
Source: Int J Obes (Lond). 2024 Jun 7;48(9):1300–6. doi: 10.1038/s41366-024-01556-w (PMC11347373; doi:10.1038/s41366-024-01556-w)
Supplement: Supplementary file 1 — Supplementary Information: Associations Between Obesity, a Composite Risk Score for Probable Long COVID, and Sleep Problems in SARS-CoV-2 Vaccinated Individuals [file 41366_2024_1556_MOESM1_ESM.pdf]

Supplementary Information: Associations Between Obesity, a Composite Risk Score for Probable Long COVID, and Sleep Problems in SARS-CoV-2 Vaccinated Individuals

Pei Xue[1*], Ilona Merikanto[2], Eva A. Delale[3], Adrijana Bjelajac[4], Juliana Yordanova[5], Rachel N.Y. Chan[6], Maria Korman[7], Sérgio A. Mota-Rolim[8], Anne-Marie Landtblom[9,10], Kentaro Matsui[11], Catia Reis[12,13], Thomas Penzel[14], Yuichi Inoue[15,16], Michael R. Nadorff[17,18], Brigitte Holzinger[19], Charles M. Morin[20], Colin A. Espie[21], Giuseppe Plazzi[22,23], Luigi De Gennaro[24,25], Frances Chung[26], Bjørn Bjorvatn[27,28], Yun Kwok Wing[6], Yves Dauvilliers[29,30], Markku Partinen[31,32], Christian Benedict[1*]

[1] Department of Pharmaceutical Biosciences, Uppsala University, Sweden
[2] Research Programs Unit, Faculty of Medicine, University of Helsinki, Helsinki, Finland
[3] Institute for Anthropological Research, Zagreb, Croatia
[4] Institute for Medical Research and Occupational Health, Zagreb, Croatia
[5] Institute of Neurobiology, Bulgarian Academy of Sciences, Sofia, Bulgaria
[6] Li Chiu Kong Family Sleep Assessment Unit, Department of Psychiatry, Faculty of Medicine, The Chinese University of Hong Kong, Hong Kong SAR, China
[7] Department of Occupational Therapy, Faculty of Health Sciences, Ariel University, Ariel, Israel
[8] Brain Institute, Federal University of Rio Grande do Norte, Brazil
[9] Department of Medical Sciences, Uppsala University, Sweden
[10] Department of Biomedical and Clinical Sciences, Faculty of Medicine and Health Sciences, Linköping University, Sweden
[11] Department of Clinical Laboratory, National Center Hospital, National Center of Neurology and Psychiatry, Tokyo, Japan
[12] Instituto de Medicina Molecular João Lobo Antunes, Faculdade de Medicina, Universidade de Lisboa, Lisbon, Portugal
[13] Faculdade de Ciências Humanas, Universidade Católica Portuguesa, Lisbon, Portugal
[14] Sleep Medicine Center, Charite University Hospital Berlin, Berlin, Germany
[15] Department of Somnology, Tokyo Medical University, Tokyo, Japan
[16] Japan Somnology Center, Institute of Neuropsychiatry, Tokyo, Japan
[17] Department of Psychology, Mississippi State University, USA
[18] Department of Psychiatry and Behavioral Sciences, Baylor College of Medicine, USA
[19] Medical University of Vienna, Postgraduate, Schlafcoaching
[20] Centre de recherche CERVO/Brain Research Center, École de psychologie, Université Laval, Quebec City, Quebec, Canada
[21] Sleep and Circadian Neuroscience Institute, Nuffield Department of Clinical

Neurosciences, University of Oxford, Oxford, UK
[22] IRCCS Istituto Delle Scienze Neurologiche di Bologna, Bologna, Italy
[23] Department of Biomedical, Metabolic and Neural Sciences, University of Modena and Reggio Emilia, Modena, Italy
[24] Department of Psychology, Sapienza University of Rome, Roma, Lazio, Italy
[25] IRCCS Fondazione Santa Lucia, Roma, Italy
[26] Department of Anesthesiology and Pain Medicine, University Health Network, University of Toronto, Toronto, Ontario, Canada
[27] Department of Global Public Health and Primary Care, University of Bergen, Bergen, Norway
[28] Norwegian Competence Center for Sleep Disorders, Haukeland University Hospital, Bergen, Norway
[29] Sleep-Wake Disorders Unit, Department of Neurology, Gui-de-Chauliac Hospital, CHU, Montpellier, France
[30] INM, University Montpellier, INSERM, Montpellier, France
[31] Department of Clinical Neurosciences, University of Helsinki Clinicum Unit, Helsinki, Finland
[32] Helsinki Sleep Clinic, Terveystalo Healthcare Services, Helsinki, Finland

* Correspondence: pei.xue@uu.se or christian.benedict@uu.se

Table of Contents

Table S1. Final sample estimation 4

Table S2. Ethical approval summary across participating countries 5

Table S3. Participant's country of residence at the time of the survey (N = 5919) 6

Table S4. Frequency of long COVID symptoms categorized by body mass index status (N = 5919) 7

Table S5. Frequency of long COVID symptoms categorized by body mass index and probable long COVID status (N = 5919) 8

Table S6. Association of BMI status with probable long COVID categorized by sex (N = 5919) 9

Table S7. Odds ratio for probable long COVID categorized by BMI status, excluding individuals with PASC scores between 3 to 11 (N = 5318) 10

Table S8. Association of BMI status with probable long COVID, excluding participants from the USA (N= 5449) 11

Table S9. Association of BMI status with probable long COVID, excluding participants with a BMI smaller than 18.5 kg/m^2 (N = 5507) 12

Table S1. Final sample estimation

**Exclusion criteria**	**N**
Participants who filled out the survey	16899
Participant underwent fewer than two SARS-CoV-2 mRNA vaccinations	-7711
Participant reported inoculation with vaccines other than mRNA **OR** participant was unclear about the administered vaccine type	-1147
Participant did not specify age	-2
Participant did not specify race/ethnicity	-1440
Participant did not specify smoking status	-98
Participant did not specify weekly physical activity level	-155
Participant did not specify main living area	-3
Participant did not specify BMI	-88
Participant did not provide information about long COVID symptoms used for scoring the post-acute sequelae of SARS-CoV-2	-114
Participant did not specify the date when the first SARS-CoV-2 mRNA vaccination was scheduled	-64
Participant did not specify nightly sleep duration **OR** nightly sleep duration was shorter than 3 hours or longer than 15 hours	-19
Participant did not fill out all questions required to estimate the insomnia severity index score	-38
Participant did not fill out all questions required to estimate the risk for obstructive sleep apnea	-5
Participant did not specify history of type 2 diabetes	-11
Participant did not specify history of attention deficit hyperactivity disorder	-1
Participant did not specify history of depression	-2
Participant's body mass index was more than three standard deviations away from the population mean	-82
**Final cohort**	**5919**

Table S2. Ethical approval summary across participating countries

Country	Ethical diary number	Notes
Austria	--	According to national research governance and regulations, the anonymous nature of survey collection did not require ethical evaluation or approval in this country
Brazil	--	According to national research governance and regulations, the anonymous nature of survey collection did not require ethical evaluation or approval in this country
Bulgaria	Protocol Number 46/05.08.2021	Ethics Commission of the Institute of Neurobiology, Bulgarian Academy of Sciences
Canada	2020-151-A-1-R-1 21-05-2021<br>REB#20-5540	The “Comité d’éthique de l’Université Laval » reviewed and approved this research protocol.<br>University Health Network Research Ethics Board, Toronto, Ontario, Canada
China (Hong Kong)	2020.277	Joint Chinese University of Hong Kong-New Territories East Cluster Clinical Research Ethics Committee
Croatia	100-21/21-4 (07.05.2021)	Ethics Committee of the Institute for Medical Research and Occupational Health
Finland	--	According to national research governance and regulations, the anonymous nature of survey collection did not require ethical evaluation or approval in this country
France	--	According to national research governance and regulations, the anonymous nature of survey collection did not require ethical evaluation or approval in this country
Germany	EA1/162/20	Ethics Committee of Charite University Hospital Berlin
Israel	AU-HEA-MK-20210603	The study was approved by the Ariel University Human Research Ethics Committee of the Faculty of Health Sciences
Italy	protocol number: 0000861, April 24, 2021	Institutional Ethics Committee of the Department of Psychology of the Sapienza University of Rome
Japan	No. 198/2020	The ethics committee of the Neuropsychiatric Research Institute, Tokyo, Japan
Norway	--	According to national research governance and regulations, the anonymous nature of survey collection did not require ethical evaluation or approval in this country
Portugal	CES-UCP nº142, May 27th, 2022	The project was approved unanimously by the Life Sciences Ethical Commission of the Portuguese Catholic University
Sweden	--	According to national research governance and regulations, the anonymous nature of survey collection did not require ethical evaluation or approval in this country
USA	IRB-20-257	Deemed exempt by the Mississippi State University Institutional Review Board.

Table S3. Participant's country of residence at the time of the survey (N = 5919)

Country	*N* (%)	Age, mean (SD)	Female sex, %
Japan	2535 (42.8)	50.2 (16.8)	46.1
Sweden	484 (8.2)	53.6 (14.2)	89.5
USA	470 (7.9)	19.4 (3.7)	74.3
Austria	323 (5.5)	44.1 (13.6)	78.6
Germany	286 (4.8)	52.0 (13.4)	43.7
Israel	272 (4.6)	34.2 (13.5)	75.7
Norway	243 (4.1)	43.0 (14.6)	74.1
Canada	214 (3.6)	50.9 (15.4)	75.2
Italy	221 (3.7)	37.6 (14.8)	76.9
Croatia	187 (3.2)	45.4 (12.6)	74.3
France	182 (3.1)	44.7 (13.5)	70.9
Bulgaria	138 (2.3)	44.2 (10.0)	84.1
China (Hong Kong)	121 (2.0)	35.9 (13.7)	67.8
Portugal	118 (2.0)	41.6 (14.5)	73.7
Finland	101 (1.7)	46.2 (13.5)	81.2
Brazil	7 (0.1)	41.4 (12.2)	71.4
Other	17 (0.3)	39.2 (11.5)	76.5

Table S4. Frequency of long COVID symptoms categorized by body mass index status ($N = 5919$)

**Long COVID symptom**	**PASC subscore**	**Normal weight, N = 3406**	**Overweight, N = 1710**	**Obesity, N = 803**	***P* value**
Post-exertional malaise	7	156 (4.6)	112 (6.5)	139 (17.3)	<.001
Fatigue	1	433 (12.7)	228 (13.3)	190 (23.7)	<.001
Brain fog	3	206 (6.0)	131 (7.7)	144 (17.9)	<.001
Dizziness	1	143 (4.2)	83 (4.9)	65 (8.1)	<.001
Gastrointestinal tract problems	1	62 (1.8)	42 (2.5)	51 (6.4)	<.001
Palpitations	2	130 (3.8)	85 (5.0)	74 (9.2)	<.001
Loss of or change in smell or taste	8	61 (1.8)	34 (2.0)	37 (4.6)	<.001
Chest pain	2	125 (3.7)	81 (4.7)	97 (12.1)	<.001

Data are presented as numbers (percentages). Group comparisons were conducted using Chi-Square tests. A significance level of $P < .05$ was used to determine statistical significance. *Abbreviations*: *PASC*, post-acute sequelae SARS-CoV-2.

Table S5. Frequency of long COVID symptoms categorized by body mass index and probable long COVID status (N = 5919)

Long COVID symptom	Participants with normal weight		Participants with overweight		Participants with obesity	
	PASC score <12	PASC score ≥12	PASC score <12	PASC score ≥12	PASC score <12	PASC score ≥12
Participants, total	3313	93	1652	58	729	74
Post-exertional malaise	76 (2.3)	80 (86.0) *	60 (3.6)	52 (89.7) *	69 (9.5)	70 (94.6) *
Fatigue	346 (10.4)	87 (93.5) *	177 (10.7)	51 (87.9) *	129 (17.7)	61 (82.4) *
Brain fog	133 (4.0)	73 (78.5) *	82 (5.0)	49 (84.5) *	79 (10.8)	65 (87.8) *
Dizziness	90 (2.7)	53 (57.0) *	45 (2.7)	38 (65.5) *	22 (3.0)	43 (58.1) *
Gastrointestinal tract problems	37 (1.1)	25 (26.9) *	20 (1.2)	22 (37.9) *	25 (3.4)	26 (35.1) *
Palpitations	79 (2.4)	51 (54.8) *	57 (3.5)	28 (48.3) *	31 (4.3)	43 (58.1) *
Loss of or change in smell or taste	31 (0.9)	30 (32.3) *	7 (0.4)	27 (46.6) *	13 (1.8)	24 (32.4) *
Chest pain	75 (2.3)	50 (53.8) *	48 (2.9)	33 (56.9) *	51 (7.0)	46 (62.2) *

Data are presented as numbers (percentages). Group comparisons were conducted using Chi-Square tests. A significance level of $P < .05$ was used to determine statistical significance. *p<0.05 indicates significance for PASC (post-acute sequelae SARS-CoV-2) score ≥12 (indicative of probable long COVID) compared to PASC score < 12 within each BMI group.

Table S6. Association of BMI status with probable long COVID categorized by sex (*N* = 5919)

Group	*N* (%)	PASC score ≥12, *N* (%)	OR [95% CI] for having probable long COVID	
			**unadjusted**	**adjusted**
Men				
Normal weight	1097 (49.4)	11 (1.0)	1	1
Overweight	828 (37.3)	8 (1.0)	0.96 [0.39-2.41]	0.56 [0.21-1.52]
Obesity	294 (13.2)	14 (4.8)	4.94 [2.22-10.99]	1.14 [0.42-3.06]
Women				
Normal weight	2309 (62.4)	82 (3.6)	1	1
Overweight	882 (23.8)	50 (5.7)	1.63 [1.14-2.34]	1.02 [0.68-1.53]
Obesity	509 (13.8)	60 (11.8)	3.63 [2.56-5.14]	1.59 [1.04-2.43]

A PASC score ≥12 was defined as having probable long COVID. Adjusted OR were derived from a logistic regression model that included the time elapsed since the first mRNA vaccination, as well as participants' age, race/ethnicity, SARS-CoV-2 test positivity, smoking status, main living area, weekly physical activity level status score, hypertension history, type 2 diabetes history, attention deficit hyperactivity disorder history, and depression history. *Abbreviations*: *PASC*, post-acute sequelae SARS-CoV-2.

Table S7. Odds ratio for probable long COVID categorized by BMI status, excluding individuals with PASC scores between 3 to 11 ($N = 5318$)

BMI group	N (%)	PASC score ≥12, N (%)	OR [95%-CI] for having probable long COVID	
			unadjusted	adjusted
Normal weight	3134 (58.9)	93 (3.0)	1	1
Overweight	1542 (29.0)	58 (3.8)	1.28 [0.92-1.78]	0.80 [0.54-1.19]
Obesity	642 (12.1)	74 (11.5)	4.26 [3.10-5.86]	2.07 [1.36-3.14]

A PASC score ≥12 was defined as having probable long COVID. Adjusted OR were derived from a logistic regression model that included the time elapsed since the first mRNA vaccination, as well as participants' sex, age, race/ethnicity, SARS-CoV-2 test positivity, smoking status, main living area, weekly physical activity level status score, hypertension history, type 2 diabetes history, attention deficit hyperactivity disorder history, and depression history. *Abbreviations*: *PASC*, post-acute sequelae SARS-CoV-2.

Table S8. Association of BMI status with probable long COVID, excluding participants from the USA (*N*= 5449)

**BMI group**	***N* (%)**	**PASC score ≥12, *N* (%)**	**OR [95%-CI] for having probable long COVID**	
			**unadjusted**	**adjusted**
Normal weight	3114 (57.1)	88 (2.8)	1	1
Overweight	1618 (29.7)	58 (3.6)	1.28 [0.91-1.79]	0.95 [0.65-1.39]
Obesity	717 (13.2)	74 (10.3)	3.96 [2.87-5.45]	1.58 [1.06-2.36]

A PASC score ≥12 was defined as having probable long COVID. Adjusted OR were derived from a logistic regression model that included the time elapsed since the first mRNA vaccination, as well as participants' sex, age, race/ethnicity, SARS-CoV-2 test positivity, smoking status, main living area, weekly physical activity level status score, hypertension history, type 2 diabetes history, attention deficit hyperactivity disorder history, and depression history. *Abbreviations*: *PASC*, post-acute sequelae SARS-CoV-2.

Table S9. Association of BMI status with probable long COVID, excluding participants with a BMI smaller than 18.5 kg/m^2 (N = 5507)

**BMI group**	***N* (%)**	**PASC score ≥12, *N* (%)**	**OR [95%-CI] for having probable long COVID**	
			**unadjusted**	**adjusted**
Normal weight	2994 (54.4)	84 (2.8)	1	1
Overweight	1710 (31.1)	58 (3.4)	1.22 [0.87-1.71]	0.94 [0.64-1.38]
Obesity	803 (14.6)	74 (9.2)	3.52 [2.55-4.86]	1.57 [1.06-2.34]

A PASC score ≥12 was defined as having probable long COVID. Adjusted OR were derived from a logistic regression model that included the time elapsed since the first mRNA vaccination, as well as participants' sex, age, race/ethnicity, SARS-CoV-2 test positivity, smoking status, main living area, weekly physical activity level status score, hypertension history, type 2 diabetes history, attention deficit hyperactivity disorder history, and depression history. *Abbreviations*: *PASC*, post-acute sequelae SARS-CoV-2.
